# How Variable Clones Build an Invariant Retina

**DOI:** 10.1016/j.neuron.2012.06.033

**Published:** 2012-09-06

**Authors:** Jie He, Gen Zhang, Alexandra D. Almeida, Michel Cayouette, Benjamin D. Simons, William A. Harris

**Affiliations:** 1 Department of Physiology, Development, and Neuroscience, University of Cambridge, Downing Street, Cambridge CB2 3DY, UK; 2 Cavendish Laboratory, Department of Physics, J.J. Thomson Avenue, University of Cambridge, Cambridge CB3 0HE, UK; 3 Institut de recherches cliniques de Montréal, 110 Pine Avenue, West Montreal, QC H2W 1R7, Canada; 4 Department of Medicine, Université de Montréal, Montreal, QC H3T 3J7, Canada; 5 The Wellcome Trust/Cancer Research UK Gurdon Institute, University of Cambridge, Tennis Court Road, Cambridge CB2 1QN, UK

## Abstract

A fundamental question in developmental neuroscience is how a collection of progenitor cells proliferates and differentiates to create a brain of the appropriate size and cellular composition. To address this issue, we devised lineage-tracing assays in developing zebrafish embryos to reconstruct entire retinal lineage progressions in vivo and thereby provide a complete quantitative map of the generation of a vertebrate CNS tissue from individual progenitors. These lineage data are consistent with a simple model in which the retina is derived from a set of equipotent retinal progenitor cells (RPCs) that are subject to stochastic factors controlling lineage progression. Clone formation in mutant embryos reveals that the transcription factor Ath5 acts as a molecular link between fate choice and mode of cell division, giving insight into the elusive molecular mechanisms of histogenesis, the conserved temporal order by which neurons of different types exit the cell cycle.

## Highlights

► Method for full live lineage tracing of retinal cells in vivo ► Demonstration that retinal clone growth is representative of retinal growth ► A stochastic model accurately predicts clone growth from equipotent progenitors ► Links between mode of cell division and cell fate help explain histogenesis

## Teaser

A key question in developmental neuroscience is how a collection of progenitors proliferates and differentiates to create a brain of the consistent size and composition. He et al. use lineage tracing to reconstruct the full retinal lineages in vivo and propose a model for stochastic control of lineage progression.

## Introduction

Most neurons are not replaced during the lifetime of the animal. Each neural progenitor, therefore, must generate a finite clone of neurons, and all these clones together must add up to the full complement of neurons in the mature nervous system. The clonal basis of vertebrate central nervous system (CNS) development has been investigated in detail in the retina, which develops from the optic cup, an outpocketing of the forebrain. The neuroepithelial layer of the optic cup is composed of retinal progenitor cells (RPCs) that first undergo a period of cellular proliferation, followed by a phase in which cells progressively exit the cell cycle. Individual RPCs are multipotent, giving rise to all retinal subtypes (Cepko et al., 1996; Holt et al., 1988; Turner and Cepko, 1987; Wetts and Fraser, 1988). In addition, clones derived from single RPCs, in a number of vertebrate species, exhibit enormous variability in both size and composition (Fekete et al., 1994; Harris, 1997; Turner and Cepko, 1987; Turner et al., 1990; Wetts and Fraser, 1988). How CNS structures, like the retina, of predictable sizes and cellular compositions arise from such variable lineages is a major unresolved question in developmental neuroscience.

The variability of clones is an intrinsic cellular feature of RPCs (Cayouette et al., 2003). This is known because isolated rat RPCs grown in vitro produce clones of various sizes and compositions. Yet, surprisingly, when examined as a population, these isolated clones are statistically similar both in size and composition to those induced in explants. As there are few extracellular influences on isolated RPCs, these results suggest that proliferation and cell fate choice are primarily determined by cell autonomous influences, such as transcription factors and components of the cell cycle (Agathocleous and Harris, 2009). What remains both controversial and unresolved, however, is whether individual RPCs use these factors within a variety of stereotyped programmed lineages or whether stochastic influences govern the expression of these factors within a population of essentially equipotent RPCs. In support of the former hypothesis, several studies have shown that RPCs exhibit cell-to-cell variability in both gene expression pattern and cell fate potential (Alexiades and Cepko, 1997; Dyer and Cepko, 2001; Jasoni and Reh, 1996; Trimarchi et al., 2008; Zhang et al., 2003). However, a recent careful statistical analysis of a set of late progenitors from the rat retina cultured at clonal density and followed in time lapse so that every division was mapped supports the latter point of view. In this study, it was revealed that the variable clone size distribution was consistent with a simple and well-constrained stochastic model in which cells were equipotent but had certain probabilities of dividing and differentiating (Gomes et al., 2011).

In many parts of the nervous system, including the retina, there is a clear histogenesis, such that some cell types tend to be born before others (Angevine and Sidman, 1961; Livesey and Cepko, 2001; McConnell, 1989; Nawrocki, 1985; Okano and Temple, 2009; Qian et al., 2000; Rapaport et al., 2004). Such histogenesis implies that, as lineages progress, the probabilities of generating distinct cell types change as a function of time or cell division. The widely accepted competence model of retinal development (Livesey and Cepko, 2001) suggests that RPCs pass through a succession of states, possibly owing to the successive expression of a set of temporally coordinated transcription factors. Indeed, homologs of temporally expressed transcription factors that orchestrate lineage progression in *Drosophila* neuroblasts (Doe and Technau, 1993) have recently been found to have similar functions in the vertebrate retina (Elliott et al., 2008). A common feature of retinal histogenesis is a substantial temporal overlap in the time windows for the generation of different cell types. In the competence model, this could be explained if the clones were not fully temporally synchronized. Recent investigations, however, show that branches or sublineages of a main lineage tree give rise to distinct cellular fates at similar or overlapping times (Vitorino et al., 2009). Single-cell sequencing studies show that neighboring progenitors at the same stage of development have many differences in their expression of cell determination factors (Trimarchi et al., 2008). These studies suggest an alternative to the competence model in which parallel sublineages may progress side by side and give rise to distinct subsets of neurons at the same time.

To gain deeper insights into these basic questions of clone size variability, stochasticity versus deterministic programming, and histogenesis at the cellular level, we developed a number of approaches to label single RPCs in zebrafish embryos and to follow these clones over time in vivo. Our results provide a complete quantitative description of the generation of a CNS structure in a vertebrate in vivo and show how a combination of stochastic choices and programmatic discrete steps in lineage progression transform a population of equipotent progenitors into a retina with the right number and proportions of neuronal types. These studies also reveal a surprising insight into the mechanism of early retinal histogenesis.

## Results

### Lineage Tracing in the Zebrafish Retina

**Figure 1:**
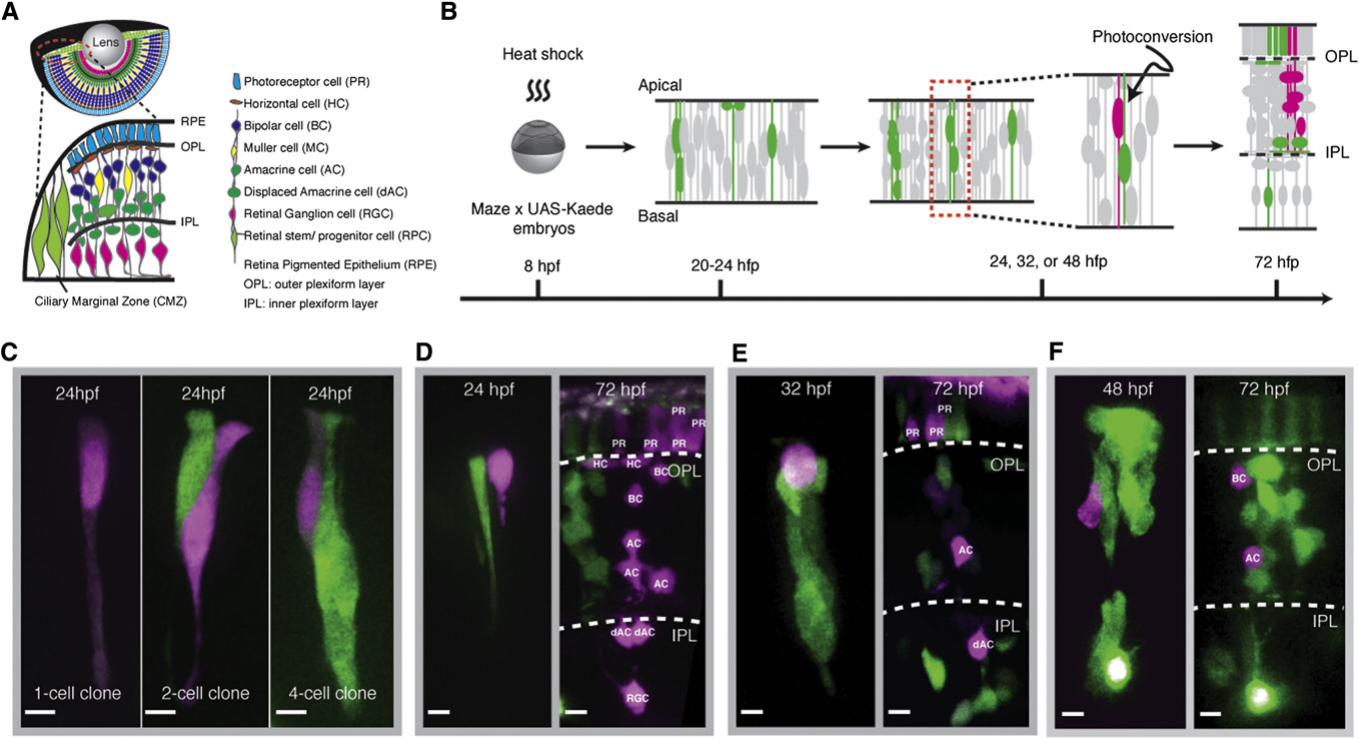
In Vivo Mosaic Labeling of Single RPCs (A) A schematic of the retina and its major cell types. (B) Experimental flow of labeling and tracking a retinal clone from a photoconverted single RPC (magenta). (C) Single RPCs (magenta) photoconverted at 24 hpf from the clones of various size (green). (D–F) Single RPCs photoconverted at 24 hpf (D), 32 hpf (E), and 48 hpf (F) (magenta, left) and resultant clones (magenta, right) with cell fates identified (Figure S1). Scale bar represents 5 μm.

To study how individual RPCs contribute to the cellular composition of the zebrafish central retina (Figure 1A), we developed a lineage-tracing method using a variation of the MAZe strategy (Collins et al., 2010). In MAZe fish, a defined heat shock is used to drive a recombinase allowing expression of Gal4, which then activates an upstream activating sequence (UAS)-driven nuclear RFP, thereby genetically marking individual progenitor cells and their progeny (Collins et al., 2010). To overcome certain limitations of this method, we used MAZe to drive cytoplasmic Kaede, a protein that irreversibly switches from green to red fluorescence upon UV exposure (Figure 1B). Fish from a MAZe line were crossed with fish from a UAS-Kaede line, and the resulting embryos were heat shocked at 8 hr postfertilization (hpf). Twelve hours later, in about 5% of such embryos, we detected either single progenitors or clones of two cells in the retina. At 24, 32, and 48 hpf, single cells in the resulting clones were randomly selected for photoconversion from green to red fluorescence (Figures 1C–1F). The red fluorescence proved to be durable enough for long-term live imaging up to 72 hpf, when the vast majority of cells in the zebrafish retina have left the cell cycle and differentiated into all the major neuronal and glial cell types (Figure 5A and see Figures S1 and S4 and Movie S1 available online).

### The Representativeness of Clones

**Figure 2:**
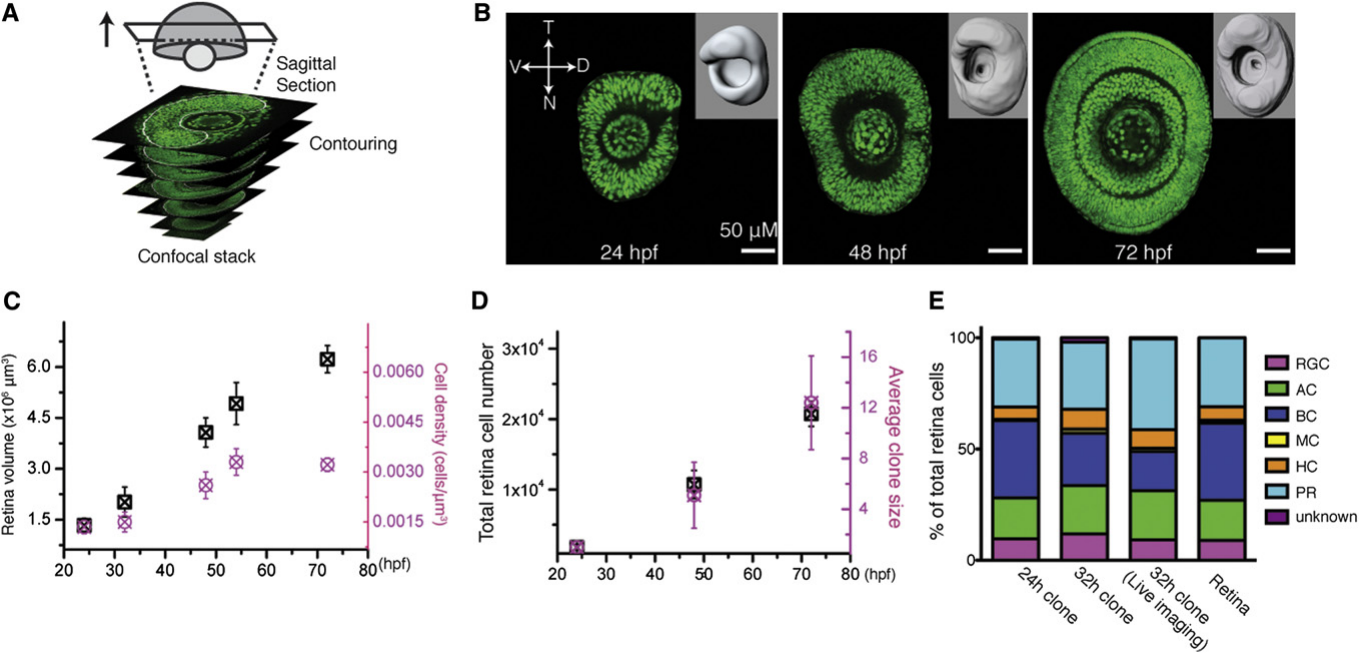
Retinal Clones Represent Retinal Growth (A) A schematic diagram of the retinal surface creation by contouring in sagittal sections (white dashed lines) of a confocal stack. (B) Representative images of the sagittal sections and the created retina surfaces (inserts) at distinct developmental stages. (C) Retinal volume (black) and average cell density (magenta) increase over time. Values are represented as mean ± SD (n = 8, 7, and 5 for the retinas at 24, 48, and 72 hpf). (D) Clone growth (magenta, photoconverted at 24 hpf) matches retina total cell growth (black) over time. Values are represented as mean ± SD (n = 8, 7, and 5 for the retinas at 24, 48, and 72 hpf). (E) Seventy-two hour postfertilization cell compositions of the clones photoconverted at 24 (24 hr clones, n = 64) and 32 hpf (32 hr clones, n = 169; 32 hr clone in live imaging, n = 67) are comparable to that of the retina.

**Figure 3:**
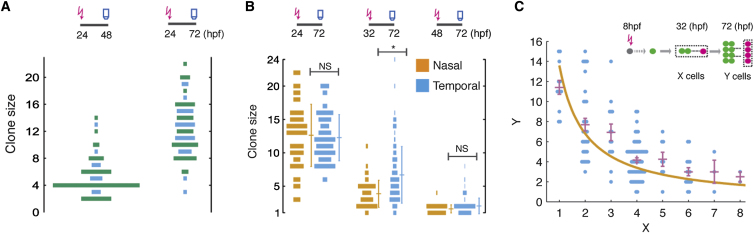
Characteristics of Retinal Clone Size Evolving over Time (A) Size distributions of the clones induced at 24 hpf and recorded at 48 hpf (left) or at 72 hpf (right), highlighting numbers of even (green) versus odd (blue) clones. (B) Size distributions of clones photoconverted at various times. The mean and SD are indicated (n = 64, 169, and 163 for 24, 32, and 48 hpf; NS, not significant; ^∗^p < 0.05, Student’s t test). Clones photoconverted at 48 hpf display significantly fewer three-cell than four-cell clones (two-proportion z test, p = 0.011). (C) After photoconversion at 32 hpf, the size of the resulting subclone at 72 hpf (y axis, depicted as the magenta cells in the schematic shown in inset) is, on average, approximately inversely correlated with the total size of the parent clone at 32 hpf (x axis, shown as the enclosed green and magenta cells in inset). The points show measurements from individual clones, while the mean and SEM are shown in purple. If the fate of RPCs is independent of clonally related cells, the average size of the subclone after photoconversion is predicted to vary as N/n where N denotes the average total clone size at 72 hpf and n denotes the average total clone size at 32 hpf. Indeed, the measured averages (purple) are broadly consistent with this prediction (orange line).

If one wishes to understand how a complete CNS structure like the retina is formed at a clonal level, it is critical to know that the growth of clones one is studying can fully account for the growth of the structure, as some marking protocols may preferentially label particular cell types or be harmful to the labeled cells. To assess whether the MAZe:Kaede retinal clones are accurately representative of retinal growth and differentiation, we first explored the growth kinetics of the whole retina. By fitting a surface to a three dimensional (3D) reconstruction of the retina, we obtained its volume at distinct developmental stages and combined this with measurements of cell density determined from confocal sagittal sections (Figures 2A–2C, Experimental Procedures, and Supplemental Experimental Procedures) to obtain total retinal cell number as a function of developmental time (Figure 2D). These results revealed that the embryonic retina consists of approximately 1,800 cells at 24 hpf (Figure 2D and Figures S2G and S2H), rising to approximately 11,000 cells at 48 hpf, and 21,000 cells at 72 hpf. This translates to a 6- and 12-fold increase, respectively. Clones derived from single progenitors at 24 hpf, as expected, showed variability in size, both at 48 hpf and 72 hpf (Figure 3A). Yet, the average increase in the size of these clones was strikingly consistent with the measured increase in total cell number in a normal retina (Figure 2D). Two other independent methods of clone induction, single-cell electroporation and transplantation, gave very similar results (Figures S2A–S2F). Moreover, clones from RPCs at 24 or 32 hpf produced, when pooled, a ratio of cell types that was comparable to the tissue’s composition (Figure 2E). These results indicate that the clones, though individually variable in size and fates, are quantitatively representative of the retina as a whole.

### The Wave of Proliferation

To investigate why retinal clones show such striking variability in size, we first looked at their size distribution as a function of time and retinal position. Clones induced from single RPCs at 24 hpf and examined at 72 hpf form a distribution that is both broad in size and independent of nasal/temporal position in the retina (Figure 3B). The distribution of clones induced at 32 hpf is also broad (Figures 3B and 3C), yet at this stage, clones positioned in the temporal zone were on average significantly larger than those derived from the nasal zone. This suggests a relative delay in the developmental program between temporal and nasal parts of the retina. Previous work has shown that a wave of differentiation progresses from central to peripheral and nasal to temporal around the zebrafish retina (Hu and Easter, 1999; Neumann and Nuesslein-Volhard, 2000). We therefore wondered whether we were observing evidence of a wave of proliferation that precedes this wave of differentiation.

**Figure 4:**
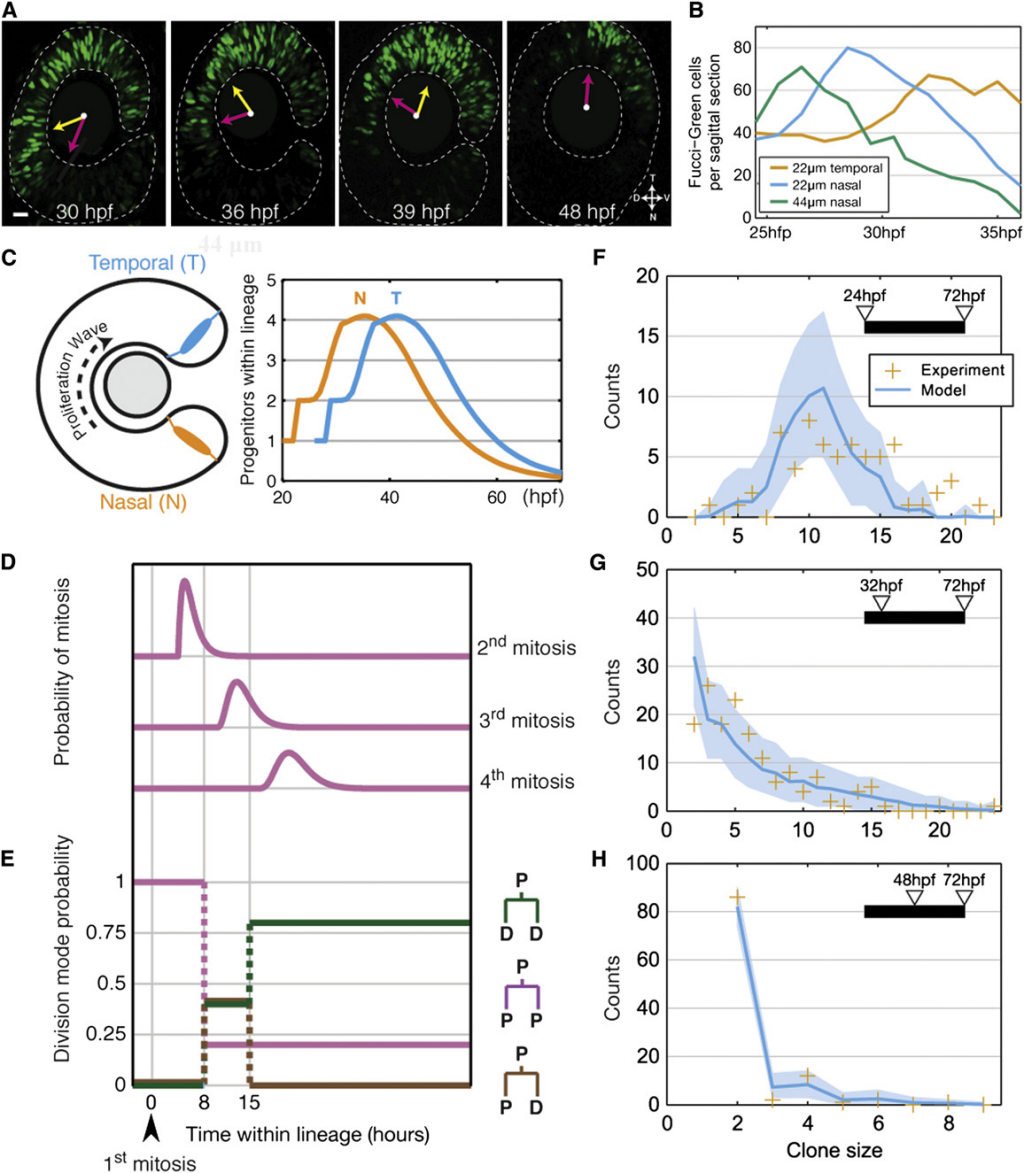
Stochasticity in Retinal Clone Growth (A) Sagittal slices of a geminin-GFP-expressing retina at various time points (yellow arrow points to region of highest number of mAG-zGem-labeled RPCs and magenta arrow points to region of geminin-GFP decline. (B) Quantified geminin-GFP-positive cells over time by zone (nasal or temporal zone) and depth (the distance between the most peripheral section and the section of interests). (C) Schematic showing the progression of the proliferative wave from the nasal region to the temporal region. On the right is plotted how the stochastic model (see Experimental Procedures) predicts the average number of progenitors derived from a single RPC as a function of time and nasotemporal position in the retina. (D) The probabilities in the model for the second, third, and fourth mitosis within a lineage to occur, measured against the first mitosis. (E) The time-dependent probabilities for modes of division of RPCs in the model. (F–H) Shows fits between model predictions (cyan lines with shaded blue regions show 95% plausible intervals due to finite sampling) and size distributions (orange crosses) of clones induced at 24 hpf (F), 32 hpf (G), and 48 hpf (H).

To study this further, we made use of a transgenic line in which actively proliferating RPCs are labeled with destabilized geminin-GFP (mAG-zGem), a marker for G2, S, and M phase of the cell cycle (Sugiyama et al., 2009). Sagittal sections revealed a wave of increasing and then decreasing green fluorescent protein (GFP)-labeled cells starting at the central nasal retina at around 23 hpf and slowly spreading peripherally and temporally (Figures 4A and 4B, Movie S2, and Experimental Procedures). By quantifying GFP-labeled RPC cell number in a fixed segment, we found that progenitors in different zones of the retina each follow the same pattern of behavior. Before the proliferation wave hits a particular region of the retina, the number of progenitors remains roughly constant. This is consistent with previous results showing that between 15–24 hpf RPCs have extremely slow cell cycle times of about 40 hr on average (Li et al., 2000). As the wave moves across the retinal primordium, RPCs transit from this near-quiescent phase to a rapidly proliferating phase with cell cycle times of 6–7 hr, whereby their number rises rapidly. After the peak of RPC proliferation, the rapid decrease in geminin-GFP signal shows that cells begin to exit the cell cycle (Figure 4B). This spreading wave, from central to peripheral and nasal to temporal, takes about 16 hr to cover the entire embryonic retina, and when it has finished, only cells in the CMZ retain geminin-GFP.

### Changing Modes of Cell Division

An individual RPC cell can either differentiate (D) or proliferate (P). For RPC numbers to increase, as at the rising phase of the proliferative wave described above, some RPCs must divide to produce two more RPC daughters, a mode of division we term PP. Similarly, for RPC numbers to decrease at the end of the wave, some RPC divisions must be terminal (termed DD). It is also possible for RPCs to divide through asymmetric PD divisions, which neither increase nor decrease RPC number. The relative proportions of these three different modes of division have been proposed to characterize other pseudostratified neuroepithelia (Simons and Clevers, 2011). To resolve the pattern of clonal evolution, we can exploit the statistical distribution of clone sizes and their evolution over time. Cell death is minimal in the developing fish retina (see below). Therefore, PD is the only division mode capable of generating odd clone sizes. We can, therefore, infer significant features of lineage progression in terms of division mode simply by examining the probabilities of clone sizes being even or odd. In particular, we noted an obvious scarcity of clones with an odd number of cells among those induced at 24 hpf and examined at 48 hpf (Figure 3A); this requires that RPCs generally proliferate by synchronized, symmetrical, proliferative PP divisions during this period. However, by 72 hpf, when all cells have exited the cell cycle, odd number clones are abundant (Figures 3A and 3B), indicating that many of these clones must at some point go through PD divisions. Finally, the scarcity of three-cell clones (especially compared to four-cell clones) among those induced at 48 hpf and examined at 72 hpf (Figure 3B) suggests that symmetric divisions also dominate the late phase of proliferation, but those divisions are differentiative (DD).

These results show that RPCs appear to go through at least three stages of decreasing proliferative capacity during development. To understand whether this is a lineage-dependent feature of RPC progression, we compared the distributions of clone sizes generated from single RPCs induced at the same developmental time in parent clones of various sizes. To do this, we induced green MAZe:Kaede clones at 8 hpf, and then we photoconverted single cells in such parent clones at 32 hpf to mark subclones in red (Figure 3C). Interestingly, we found that the larger the parent clone, the smaller, on average, the subclone. For example, subclones of two-cell parent clones are, on average, about eight cells, whereas subclones of eight-cell parent clones are, on average, only about two cells (Figure 3C). This inverse proportionality shows that RPCs intrinsically lose proliferative potential as clones grow. However, what is remarkable is that the spread of subclone sizes is large in all cases. For example, subclone sizes from two-cell parent clones are as large as 15 and as small as three (Figure 3C). This variability of subclone sizes within lineages seems difficult to reconcile with any simple deterministic instructions of parent RPCs.

### A Stochastic Model of Cell Division Mode Predicts Clone Size Distributions

These findings point to a developmental program in which a wave of symmetrical proliferation (PP) followed by asymmetrical (PD) and then terminal (DD) differentiative divisions spreads around the retina. However, if all RPCs at 24 hpf went through exactly the same program (e.g., two rounds of PP to produce four P cells, followed by one round of PD to produce four D and four P cells, followed by one round of DD), all clones would end up being exactly the same size, i.e., 12 differentiated cells. This would generate a retina of approximately the right total number of cells. However, such a stereotypic pattern of RPC lineage progression is not consistent with the large variability in clone sizes of 24 hpf RPCs. As a stochastic model provides an excellent fit to clone size distribution for rat retinal progenitors grown at clonal density in vitro (Gomes et al., 2011), we asked whether a similar model would be useful in predicting clone size distributions of zebrafish retinal clones in vivo. Using the proliferation wave to estimate the timing of the transitions from PP to PD to DD, and the average cell-cycle length, we developed a simple computational model (Figures 4C–4E and Experimental Procedures). In this model, once activated by the proliferative wave, RPCs transfer between a phase of symmetrical cell division (PP) to a narrow phase in which all three modes of division (PP, PD, and DD) coexist with fixed probabilities. The final phase is one in which cell divisions are predominantly terminal (DD). RPCs at the same stage of lineage development are presumed to be equipotent in terms of their proliferative potential. The stochastic element means that it is a matter of pure probability whether RPCs divide according to one mode or the other. Previous history, except for the fact that D cells can no longer divide, is presumed to play no role. Thus, for example, a PP division could follow a PD division within the stochastic window. The final phase is one in which cell divisions are predominantly terminal (DD). By estimating only the time window during which PP, PD, and DD divisions were concurrent, and the probability of PD division within that time window, this simple stochastic model predicts experimental clone size distributions over a range of time points with striking precision (Figures 4F–4H).

### Live Imaging of Clones

**Figure 5:**
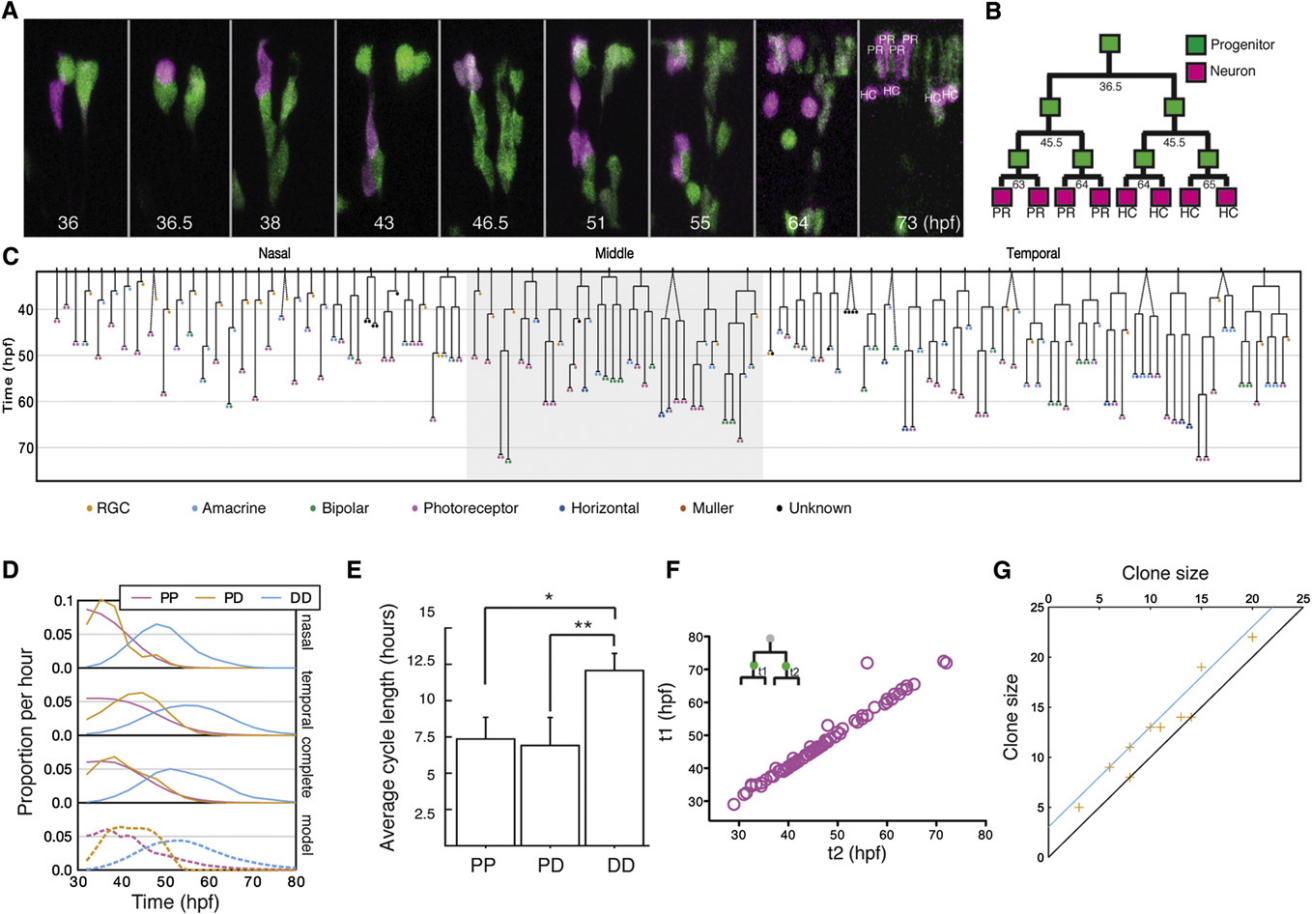
In Vivo Time Lapse of Retinal Clone Development (A) An image series of the generation of an eight-cell clone from a single RPC photoconverted at 32 hpf (magenta). (B) The schematically reconstructed lineage tree for the clone in (A). (C) Summary of 60 complete retina lineages induced at 32 hpf reconstructed from in vivo live imaging. Dashed lines indicate clones in which the first division happened between photoconversion and start of time lapse. (D) The normalized rates of different division modes evolve over time, derived from the lineages recorded in (C), compared to model prediction (dashed lines in bottom panel). (E) The bar graph shows the length of cell cycle that leads to the three division modes. PP, symmetric proliferation; PD, asymmetric differentiation; DD, symmetric differentiation. Values are represented as mean ± SEM (n = 28, 16, and 118 for PP, PD, and DD, respectively; ^∗^p < 0.05, ^∗∗^p < 0.05; Student’s t test). (F) The division time of sister RPCs (t1 and t2) within the time window of 24 and 72 hpf, indicating the synchrony of sister divisions. (G) The correlation between sizes of sister lineages (orange crosses) compares well with the expected correlation due to synchronization of second mitosis induced by proximity in space and time of sister RPCs (cyan).

We next asked whether this model could predict the division patterns actually observed in a population of single clones in vivo. To this end, using the MAZe-Kaede method coupled with four dimensional (4D) confocal microscopy, we were able to acquire 24 time-lapse movies of single cell-derived clones induced at 24 hpf and followed until 48 hpf (Figure S4I) and 60 movies from 32 hpf to 72 hpf (Figure 5C, Figures S4A–S4F, and Movie S3). In these movies, every cell division and differentiation event can be reconstructed (Figure 5C). This ensemble of clones was also fully representative of retinal growth (Figure S2F). As only 1.5% of cells died during our time-lapse movies, cell death is not considered to be a major factor in generating a retina of the correct size and neuronal composition. As predicted by the model, the reconstructed lineages confirm that the vast majority of early cell divisions were symmetric and proliferative (PP) (Figures 5A–5C and Figure S4I) and that by 32 hpf, the proliferation wave had passed through much of the nasal retina, leaving it in a differentiating phase (Figures 5C and 5D). The live-imaging data also show a clear predicted phase of clonal development in which all three modes of division, PP, PD, and DD, are simultaneously present at intermediate times. Finally the predicted terminal phase of DD divisions (Figure 5D) is confirmed by the live-imaging data. We also find, as the model predicts, several instances in which PP divisions follow PD divisions within clones. The success of this model strongly favors the hypothesis that RPCs are equipotent in terms of their proliferative potential but subject to stochastic influences.

The live-imaging data also allowed us to measure directly the average and distribution of cell cycle times, separated according to outcome. In this case, we find that both symmetrical PP-type cell divisions and asymmetrical divisions are narrowly distributed around a similar average of 7.5 ± 1.3 hr (Figure 5E), consistent with recent direct measurements of the cell cycle in the zebrafish retina at these stages (Baye and Link, 2007; Leung et al., 2011). However, terminal DD-type divisions have cell cycle times of 12.1 ± 1.0 hr (Figure 5E)—a feature that does not impact on the measured clone size at 72 hpf when the retina is complete. Another feature of the live-imaging data is the finding that, over the developmental time window, sister P cells show highly correlated cell cycle times (Figure 5F). In terms of the sizes of clones they generate, however, sister RPCs show no more correlation than one would expect from the model based simply on the synchronization of consecutive mitoses coupled with the proximity in space and time of sister RPCs (Figure 5G). These data are consistent with the equipotent stochastic model and argue that RPCs, if programmed at all, are not programmed in such a way that sister sublineages behave as twins.

### Clonal Histogenesis and Cell Fate

**Figure 6:**
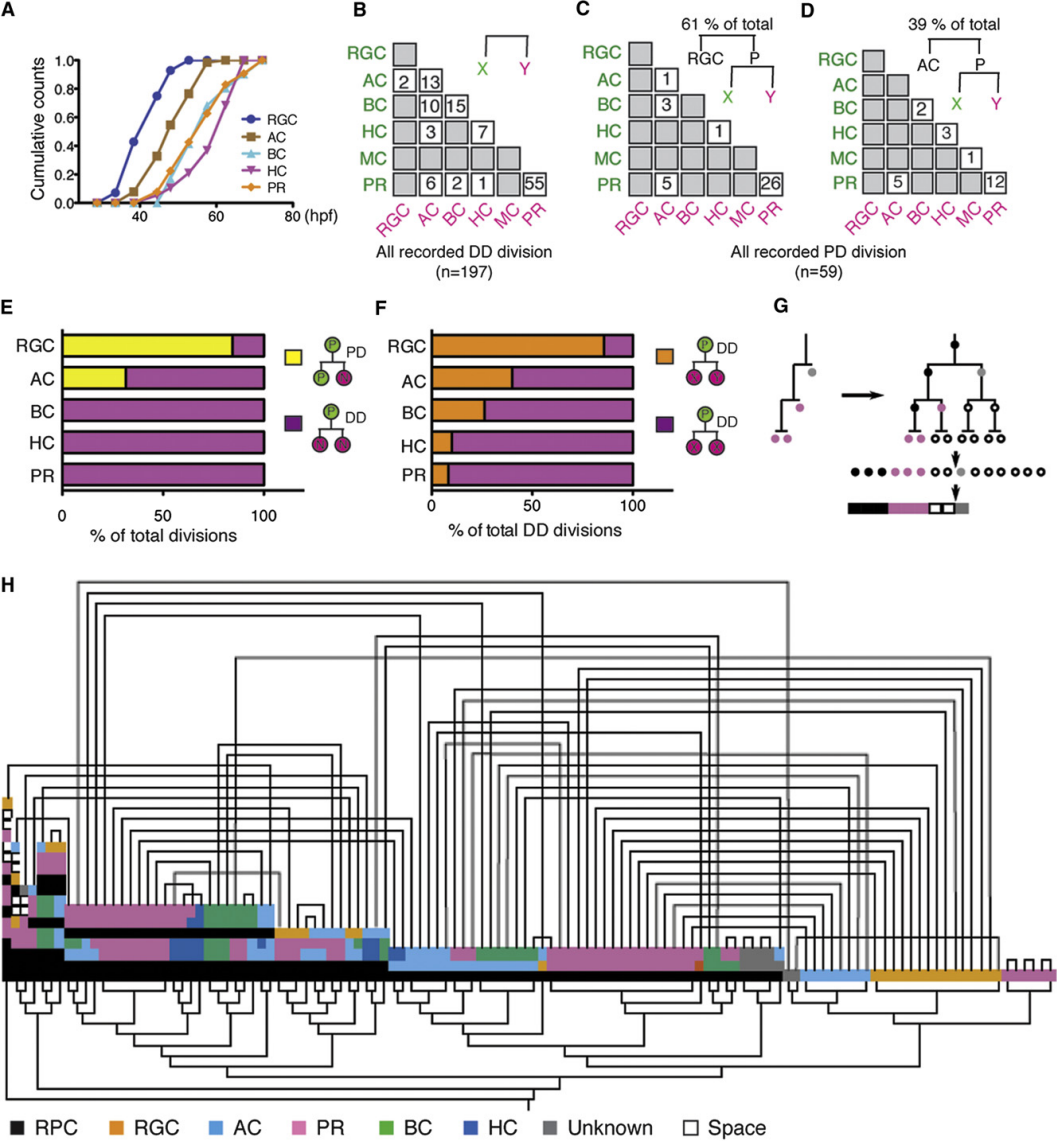
Clonal Histogenesis (A) The histogenesis of retina cells of different types derived from the live-imaging data. (B) Matrix summarizing the cell composition of 197 DD divisions. (C and D) The fate outcomes of the P progenitors of 59 PD divisions, 36 from cases in which the D cell was an RGC (C) and 23 in which it was an AC (D). (E) Bar graph showing the proportions of the PD and DD divisions in generation of distinct cell fates. (F) Bar graph showing the proportions of symmetric DD divisions (XX) and asymmetric DD divisions (XY) in generation of distinct cell fates. (G) Illustration of compressing of single lineage tree into a barcode. (H) Barcode cluster analysis of clones from (C) split into sister lineages (connected above), embedded into the smallest symmetric tree (inserting space as necessary), and converted into a barcode by a depth-first traversal to preserve structural units and hierarchically clustered according to Levenshtein distance (shown by the tree below). While sisters show similar sizes (due to their being born at the same time and place), there are no other obvious correlations between sister lineages.

An unresolved issue in retinal development is how histogenesis, the fact that some types of cells tend to be born before other types, is expressed within individual lineages. This is because, at the population level, several different cell types are often born within the same time window (Belecky-Adams et al., 1996; Holt et al., 1988; Nawrocki, 1985; Rapaport et al., 2004). These periods of overlap could indicate poor synchronization of RPCs that are all intrinsically programmed to go through a strict histogenetic process in line with a competence model, or it could be that individual lineages can generate different cell types at the same time. The live-imaging data allow us to address this question directly. By combining data from multiple lineages, we first show that the histogenesis of cell types (Figure 6A) matches well with previous birthdating studies in zebrafish using DNA labeling methods (Jusuf et al., 2011; Nawrocki, 1985). At a clonal level, we see that a neuron of one type can have as its simultaneously born sister almost any other type of neuron (Figure 6B). Indeed, in several lineages, three different cell types are born within minutes of each other (Figure 5C). These facts imply that within a clone, there is no strict order of successive competence. Rather, the overlapping order of retinal histogenesis seen in cell population birthdating analyses (Holt et al., 1988; Nawrocki, 1985; Rapaport et al., 2004; Young, 1985) is an inherent feature of the variability of histogenesis within single clones.

What then is the nature of clonal histogenesis? First, we find that RGCs tend to arise from the differentiating D daughter of a PD division during the brief phase of asymmetrical (PD) divisions (Figures 5C and 6E). ACs, the next cells to be generated, are derived at a time when both PD- and DD-type divisions compete (Figures 6B, 6D, and 6E). The remaining cell types, BCs, HCs, and PRs, appear, on average, later in development when all divisions are terminal, DD (Figures 6B and 6E), and show a heavy weighting toward symmetrical fate outcomes (Figure 6F).

If RPCs are equipotent not only with respect to proliferative potential but also with respect to cell fate choice, then different fate choices should be available to all RPCs at any time, with the probabilities of each fate changing during clonal progression, in line with global histogenesis. To see whether this is the case, we used a barcode cluster analysis of clones by lineage similarity (Figures 6G and 6H). This analysis shows more than 30 different species of lineage in terms of clone size, cell fate, and division pattern. Among these, other than HCs, BCs, and PRs, which generally appear as terminal pairs, there is no greater chance that two sister RPCs will have related or mutually predictable lineages than nonsister pairs generated at the same time and position. This finding is consistent with stochasticity of fate choice among equipotent RPCs within the loose constraints of clonal histogenesis and argues against any programming of RPCs such that early sister lineages produce clones of the same size or composition.

### Ath5 Links Mode of Division and Cell Fate, Providing Insight into Histogenesis

**Figure 7:**
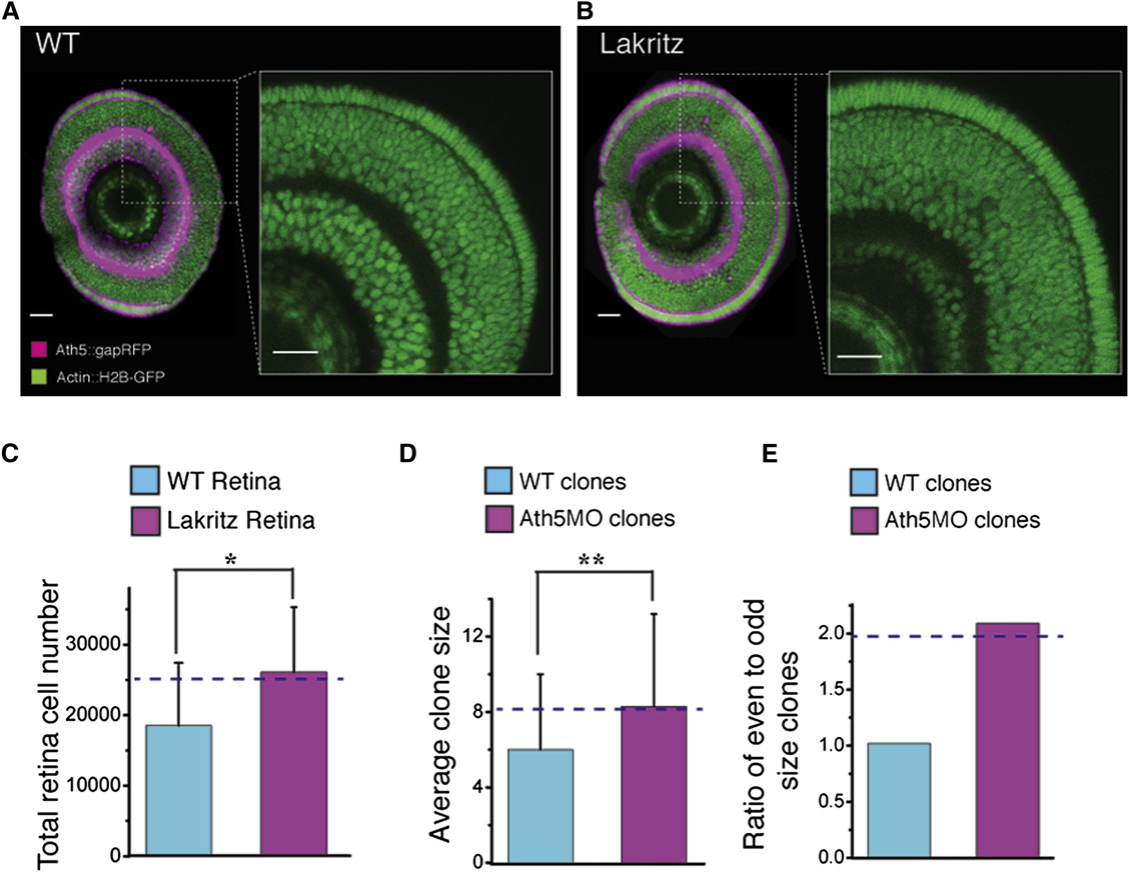
Total Retinal Cell Number Increase in Ath5 Mutants Predicted by Modeling (A and B) Zoomed-in images of a retina sagittal section of wild-type (WT) (A) and *lakritz* (B) retina, showing cell number increase in the *lakritz* retina. Scale bar represents 23 μm. (C and D) Quantified increase in total cell number in the *lakritz* retina (C) and the average clone size in Ath5 morpholino (Ath5MO)-injected retinas (D). The dashed lines represent the model prediction. Values are represented as mean ± SD (n = 4, WT retina; n = 3, *lakritz* retina; n = 169, clones in the WT retina; n = 34, clones in the Ath5MO-injected retina; ^∗^p < 0.05, ^∗∗^p < 0.05, Student’s t test). (E) Ath5MO-injected clones are biased toward even numbers, as predicted by the model (dashed line).

The link between RGC fate, which marks the start of many retinal lineages, and the PD mode of division suggests that the bHLH transcription factor Ath5 (Atoh7), which is necessary for the generation of RGCs (Kanekar et al., 1997; Kay et al., 2001), might also be involved in the mode of cell division. Ath5 is expressed in some RPCs prior to a differentiative division generating an RGC (Poggi et al., 2005). Our results show that in 80% of the cases, the other daughter of this division is a progenitor cell that divides again (Figure 6E). A previous study indicates that in *lakritz* mutants (in which the *ath5* gene is mutated), there is a delay in differentiation by the equivalent of approximately two cell cycles (Kay et al., 2001), suggesting that the cell that would have become an RGC effectively reverts back to the fate of its parent to undergo a PP rather than a PD division. Such reversions back to the parental lineage have been seen in *unc-86* mutants in *C. elegans* (Chalfie et al., 1981). Incorporating such a scenario into our stochastic model of clone size evolution, we expect to see that MAZe-Kaede clones as well as the total cell number in *lakritz* or *ath5* morphant retinas would, on average, be 35% larger. In striking agreement with this prediction, the experimental results show an increase of 40% in clone and retinal size (Figures 7A–7D). Moreover, the conversion of PD-generating RGC divisions to PP divisions biases Ath5 morphant clones toward even numbers by an amount that is in good agreement with the model prediction (Figure 7E). This dual function of Ath5 in RGC fate and early PD cell cycle exit within clones not only strongly supports our stochastic model, but it also provides a mechanistic insight into the first step in retinal histogenesis, the early birth of RGCs.

## Discussion

In the late 1980s, newly developed methods of clonal analysis in vivo revealed that retinal progenitors were multipotent (Holt et al., 1988; Turner and Cepko, 1987; Wetts and Fraser, 1988). This initial insight led to many questions that have still not been resolved, such as (1) why are some clones bigger than others; (2) what are the mechanisms by which clonally related cells choose different fates; and (3) is there a strict order of cell genesis within clones? To address these important questions, it is obviously useful to see full clones grow and differentiate into mature neurons in real time in the CNS in vivo. Until recent improvements in imaging and genetic labeling strategies, however, this has not been possible. Using a variation of the MAZe strategy (Collins et al., 2010) in combination with 4D microscopy, we have been able to label single progenitors at precise stages and follow their development in time lapse until all their progeny have differentiated into specific neuronal types that we could unambiguously categorize.

The variability of clone size and composition, seen here and in all previous retinal studies (Holt et al., 1988; Turner and Cepko, 1987; Turner et al., 1990; Wetts and Fraser, 1988; Wong and Rapaport, 2009), raises a key question about whether RPCs have individually fixed lineage programs, like *Drosophila* CNS neuroblasts, or whether they are a set of equipotent progenitors subject to stochastic influences. There is good evidence for the heterogeneity of RPCs at neurogenic stages, in particular, in respect to gene expression patterns (Alexiades and Cepko, 1997; Dyer and Cepko, 2001; Jasoni and Reh, 1996; Zhang et al., 2003), and it is possible that these differences account for the variety of lineage outcomes. No experiment can absolutely rule out that the heterogeneity of clones follows from the individual and early specification of RPCs, just as no finite sequence of numbers can be proved to be part of nonrandom series. Nevertheless, in our data set, the very large variety of clone types, in size, composition, and division pattern, and particularly the variability among subclones and sister clones, seems hard to reconcile with detailed deterministic programming. Most importantly, the data presented here, at least in relation to clone size, are consistent with a very simple and constrained stochastic model operating on equipotent RPCs when tested against every statistical measure. One might therefore wish to consider the possibility that many of the molecular differences seen in RPCs may not be programmed but rather are the result of cycling or stochastic fluctuations in gene expression (Elowitz et al., 2002; Hirata et al., 2002; Munsky et al., 2012).

Similar models of stochastic proliferation have been very successful at predicting the lineages of progenitors in homeostatic self-renewing adult tissues in vivo (Clayton et al., 2007; Klein and Simons, 2011). A recent analysis of clones generated in vitro from late-stage rat RPCs shows that simple stochastic rules similar to those uncovered here, but with different probabilities of PP:PD:DD, are very powerful in predicting the size distribution of these clones (Gomes et al., 2011). While this model provides an excellent fit with clone size distributions seen in the zebrafish retina in vivo, it was designed specifically for clone size rather than cell fate distributions. The data set we have is simply not sufficient to allow us to generate a useful model of cell-type distributions within clones, although in the future, with advances in imaging, this should become possible. While the variability of clonal compositions generated by sister RPCs strongly suggests that there are likely to be stochastic elements at work in terms of fate assignment, there are also several clear trends in the data that show cell fate determination is unlikely to be purely stochastic. For example, the frequency of same-type pairs of PRs, HCs, BCs, and ACs is much higher than one would predict from a purely stochastic model, as is the probability that the sister of an RGC will be a P cell.

A pervasive feature of the development of many CNS tissues is histogenesis, the general ordering of cell type by birthdate. For example, the cerebral cortex famously shows an inside-out histogenesis, and this order of cell birth is intrinsic to progenitors, as when grown at clonal density in vitro, they give rise to clones in which there is a distinct general order of cell-type production (Qian et al., 2000). However, it is unknown why layer VI cells exit the cell cycle before layer V cells, etc. Similarly, in the retina, RGCs are born first in a variety of vertebrate species. Why should this be so? Previous studies have provided important hints about these questions by showing that temporal identity genes, homologous to those identified in *Drosophila* neuroblasts, might also act as fate-biasing factors in RPCs to increase the probability of adopting certain fates associated to a particular temporal window (Elliott et al., 2008), but such genes have not been shown to cause early cell cycle exit. Other studies show that some cell-type determination factors may also lead to cell cycle exit and vice versa (Ohnuma et al., 1999, 2001), but their timing of expression does clearly coincide with cell birthdate. It is therefore challenging to ascertain how these factors work within the context of histogenesis, especially when stochastic mechanisms appear to influence cell cycle exit and fate choice. The finding that Ath5, already known to be essential for RGC cell fate, is also involved in early PD divisions leading to cell cycle exit at the initiation of retinal clones thus sheds mechanistic insight into how histogenesis can be accomplished within a stochastic system.

In summary, we have shown that the generation of the zebrafish retina can be accurately described by a combination of stochastic and programmatic decisions taken by a population of equipotent RPCs. As these cells move through the lineage program, the stochastic model accurately describes how these cells generate clones of variable size, and it also accurately predicts many characteristics of the actual lineage trees that we have seen in our time-lapse studies. Stochasticity also appears to be a feature of cell fate assignment. We therefore speculate that all vertebrate retinas, though vastly different in size and the proportional composition of different cell types, may follow similar stochastic rules but tune their proliferative and cell fate probabilities to arrive at appropriate species-specific retinal sizes and cellular compositions.

## Experimental Procedures

### Animals and Transgenic Lines

Zebrafish lines were maintained and bred at 26.5°C. Embryos were raised at 28.5°C and staged in hours postfertilization (hpf). Embryos were treated with 0.003% phenylthiourea (PTU, Sigma) at 8 hpf to delay pigmentation and were anaesthetised by 0.04% MS-222 (Sigma) prior to live imaging. All animal work was approved by Local Ethical Review Committee at the University of Cambridge and performed according to the protocols of UK Home Office license PPL 80/2198. Geminin-GFP (Tg(EF1α:mAG-zGem(1/100))^rw0410h^), UAS-Kaede, and MAZe transgenic lines have been described previously (Collins et al., 2010; Scott and Baier, 2009; Sugiyama et al., 2009). H_2_B-GFP transgenic line was generated by injection of the actin promoter-driven H_2_B-GFP DNA construct.

### Cell Number Estimation in Retina

The cell number of entire retinas or individual cell types was formulated by multiplying the cell density by the volume of retinas (or individual cell layers). To measure the volume, we acquired the confocal z stacks of entire retinas at distinct stages (24, 32, 48, 52, and 72 hpf) on the inverted confocal microscope (Olympus FV1000) equipped with 40× oil objective (NA = 1.3). The surfaces of retinas were created based on retinal confocal stacks using the contouring adaptive tools in Imaris 7.3 (Bitplane). To distinguish different cell layers, we crossed the H2B-GPF line with the Ptf1a-DsRed line, in which all layers were separated in space by the Ptf1a-DsRed labeling and the surface of individual layers therefore could be reliably generated. The resultant surface was further used to calculate the volume using the statistics tool in Imaris 7.3.

Cell density was estimated by counting the number of cells in given 1 μm sagittal section acquired using the confocal microscope (Olympus FV1000), at a depth in which all the cell layers were present, followed by a necessary correction using the protocol outlined in Figure S7. Cell number in retina sections (or individual cell layers) was counted manually using ImageJ or Photoshop CS5 (Adobe), and the corresponding areas were measured using the contouring adaptive and statistics tools in Imaris 7.3.

### In Vivo Single-Cell Electroporation

Twenty-four hour postfertilization embryos embedded in 1% low-melting agarose (type IV, Sigma) were prepared in the Steinberg’s solution (100× stock: 0.5 g KCl, 0.8 g Ca(NO_3_)_2_ × 4H_2_O, 2.1g MgSO_4_ × 7H_2_O, 34 g NaCl, 119 g HEPES, to 1 l dd H_2_O [pH 7.4]) with MS-222 and PTU in a customer-made imaging chamber. The single-cell setup for Axon Axoporator 800A Electroporator (Molecular Devices) was wired as described previously (Bestman et al., 2006; Haas et al., 2001). An electrode with a 1 μm opening (20–50 MΩ resistance) was pulled using a micropipette puller (Model P-97, Sutter Instrument) and back filled with 1 mM Dextran Alex 488 dye (Invitrogen). The retinal region of interest was found using an upright compound fluorescent microscope equipped with 40× water objective (NA = 0.8). When the electrode tip touched the desired cell, a negative voltage square pulse was applied (200 Hz, 500 ms train duration, 2 ms pulse duration, 5V). A single retina RPC could be visualized instantly in green upon a successful electroporation. The electrode stayed for at least 20 s before slow and careful withdrawal to avoid cell damage. Embryos were then removed and raised in embryo medium for further analysis.

### Heat Shock and Photoconversion

The MAZe line was crossed with the UAS-Kaede line. Embryos were collected and kept at 28°C. At 8 hpf, a brief heat shock was applied at 39°C for 1 min. After 12 hr, the heat-shocked embryos were screened on an upright fluorescent microscope and the retinas with Kaede-expressing cells were selected. At 24, 32, or 48 hpf, embryos were embedded in 3% methylcellulose (Sigma) and the green clones were found using a 60× water objective (NA = 1.3) on the spinning-disc microscope (Perkin Elmer). Single cells from the green clones were then randomly targeted and photoconverted by applying a 5 s train of 405 nm laser pulses.

### Transplantation

H_2_B-GFP transgenic or wild-type embryos with fluorescent protein mRNA injection at the one-cell stage were used as donors. At the blastula stage (4 hpf), the embryos were dechorionated by 0.3 mg/ml pronase and positioned in the custom-made transplantation mold. Less than five donor cells were transplanted into the animal pole of host embryos, where the cells are expected to develop into retina cells (Kimmel et al., 1990). The host embryos were then recovered at 32°C for 2 hr before being returned to 28.5°C and screened on an upright fluorescent microscope at 24 hpf to select those with one- or two-cell retinal clones.

### Confocal Image Acquisition and Analysis

Embryos at desired developmental stages were collected and embedded in 3% methylcellulose with the proper orientation. Retina clones or entire retinas were imaged under 40× oil (NA = 1.3) or 60× silicon (NA = 1.35) objectives on the inverted laser-scanning confocal microscope (Olymus FV1000). All the images were acquired by the comparable setting (1,024 × 1,024 resolution, 10 μs/pixel scanning speed, 1–1.2 μm optical section). Image analysis was performed using ImageJ or Volocity software (Improvision).

### In Vivo Live Imaging

Dechorionated embryos were collected at desired time points, such as 24 or 32 hpf. After screening and photoconversion, embryos were embedded in 1% low-melting agarose in the customer-made imaging dish. Four-dimensional live imaging was conducted on the inverted laser-scanning confocal microscope (Olympus FV1000) at 28.5°C controlled by a customer-made heating block. For 24–48 hpf time lapse, image stacks were acquired every 30 min. And for 32–72 hpf time lapse, images were acquired every 30 min for the first 12 hr and every 60 min afterward. Three-dimensional stacks of retina clones provided the necessary image resolution with the minimum laser exposure. Four-dimensional movies were analyzed using Volocity software.

### Modeling: Clone Size and the Proliferation/Differentiation Wave

To characterize the evolution of RPC fate during retinal development, we combine lineage-tracing measurements with geminin-GFP data to define the simplest cell kinetics consistent with the experiment. The validity of this modeling scheme can then be assessed and challenged by live-imaging studies.

The moderate variability in size of clones at 72 hpf induced at 24 hpf indicates a role for stochasticity in controlling the balance between proliferation and differentiation (Figure 3B). However, the correlation between the size of a clone at 32 hpf and the eventual size of the clone (Figure 3C) and the proliferative/differentiate wave implies that, within each lineage, there is a progression of stochastic probabilities, indexed against an internal clock. In particular, we can rule out a precisely and internally specified program of development, as well as a time-invariant process of equipotent progenitors (such as would exist in the CNS during adulthood).

Therefore, we will suppose that RPCs form a functionally equivalent, equipotent cell population with evolving proliferative potential, which is decoupled from the particular specification of individual cell types. Through temporal and spatial correlations, we expect to capture many aspects of the data, including correlations that might otherwise require a causative hypothesis. Any residual correlations between lineage and clone size are therefore a reflection of the histogenesis of cell types or a signature of early fate specification.

In this paradigm, RPCs follow a (stochastic) developmental program, passing from a near-quiescent phase to an active proliferating phase and finally to a differentiating phase. The initiation and timing of this developmental program is defined by the wave of proliferation that sweeps around the retina, starting at the central nasal region and terminating at the peripheral temporal zone. In the following, we will use the timing of the first mitosis to define the start of the development program within each lineage. This occurs at around 23 hpf in the central nasal region, reaching the peripheral temporal region around 16 hr later. For simplicity, we therefore suppose that RPCs enter their active phase at a uniform rate, expecting that deviations from this will be beyond the resolution of the data.

If we assume that, over the period from 24 to 48 hpf, RPCs are limited to the proliferative phase, measurements of the average clone size over this period suggest a cell cycle time of ca. 6 hr, allowing approximately two rounds of symmetrical cell division. (Anticipating the results of the live-imaging study, our simulations are actually performed with a shifted gamma distribution, with a refractory period of 4 hr, mean of 6 hr, and width of 1 hr.) In addition, the lack of odd-sized clones (Figure 3A) requires a high degree of synchrony between division times of sister progenitors; we assume a difference between sister cell cycles of around 1 hr, normally distributed. Moreover, since the average clone size grows 12- to 13-fold over the period from 24 hpf to 72 hpf, we can deduce that each progenitor at 48 hpf must go on to produce, on average, three postmitotic cells. Thus, we may visualize a “typical” clone to consist of two rounds of symmetrical (PP-type) division, one round of asymmetrical (PD-type) division, and one round of terminal (DD-type) division leading to the average 12-fold increase in average clone size over the time course.

However, the variability in size of clones at 72 hpf, induced at 24 hpf, provides a strong signature of stochasticity in cell fate choice. We therefore suppose that, within a lineage, the balance between proliferation and differentiation is achieved through stochastic fate decisions, with probabilities that vary through the developmental stages (Figure 4E). For simplicity, we assume these changes to occur instantaneously, thus avoiding having to parameterize the change beyond just a single time. In particular, since clones induced at 48 hpf involve very few three-cell clones, PD divisions must be suppressed at these later times. Thus, there must be at least two such changes, to start and then stop PD divisions; we assume that there are only these two. Indeed, the proportion of four-cell to two-cell clones (Figure 3B) suggests that one in five cell divisions involves symmetrical self-renewal, while the remaining four divisions are terminal.

Thus, to fully define the model, we only have to specify two time points to delineate the intermediate PD phase and the probabilities within that phase. The times were chosen to be 8 hr and 15 hr after the first mitosis, which essentially straddle the subsequent bursts of mitoses; it was found that the outcome was not particularly sensitive to the precise timing in any case, as long as they did not significantly reassign mitosis to be in different phases. The proportion of PP divisions was chosen, for simplicity again, to be the same as the terminal phase, i.e., one in five. The final parameter, the probability for PD divisions, was chosen to give the correct average size of 72 hpf clones induced at 24 hpf, which corresponded to two in five divisions. The proportion of DD is thus two in five during this intermediate phase.

This model was implemented as a custom-written Monte Carlo simulation, which outputs probabilities for observing clones of different sizes. Figure S3 shows how variation in the parameters affects the model output. While a comparison of the measured clone size distribution to the model reveals a favorable fit to the experimental data (Figures 4F–4H), the freedom to adjust control parameters limits its credibility. Fortunately, we can make use of the live-imaging data to challenge some of the assumptions and predictions of the model. This comparison is discussed in the main text.

### Barcode Analysis of In Vivo Lineage Tracing

To answer the question of whether fate choice is specified early on, we undertook an analysis of sister lineages from clones in the reconstructed in vivo live imaging. Although rudimentary, it is somewhat quantitative. In particular, we compress each subclone from a tree into a string (represented graphically as a bitmap in Figure 6G) and compare strings by a standard Levenshtein distance measure (which counts the number of single-character edits that would be necessary to turn one string into another). Finally, we use a standard hierarchical clustering algorithm to sort the strings according to their similarity.

It was important to compare not only the final cell types generated by each lineage but also the structure and order in which the cells appear. To do this, we chose a particular representation of trees as strings in order to preserve the tree structure. Specifically, we embeded each tree into a complete tree of sufficient depth, then performed a depth-first traversal to gather the cell types into a string (Figure 6G).

Figure 6H shows the subclones from the live-imaging data (Figure 5C), with hierarchical similarity shown as a tree at the bottom and sister lineage relation at the top. We can discern no significant patterns from this data.

## Supplemental Information

Document S1. Figures S1–S4 and Supplemental Experimental Procedures

Table S1. List of Clones

Movie S1. Sister Clones in 3D Three-dimensional animation of a pair of retina sister clones (in green and magenta) at 72 hpf, which are generated by photoconversion of a single retina progenitor cell from a Kaede-expressing two-cell clone at 24 hpf. Scale bar represents10 μm.

Movie S2. mAG-zGEM in 3D Three-dimensional animation of the retina with the expression of mAG-zGem (green) and H2B-RFP (magenta, mRNA-injected at the one-cell stage) at 24 and 40 hpf, showing retina nasal zone expands its size prior to the temporal zone, while the nasal progenitors are leaving the cell cycle (indicated by the loss of mAG-zGem) earlier than those from the temporal zone. Scale bar represents 25 μm.

Movie S3. Time Lapse of Eight-Cell Clone Time lapse of an eight-cell clone (in Figures 4A and 4B) developed from a single RPC (magenta) from 35 hpf throughout 73 hpf.
