# Supplementary material for: How Variable Clones Build an Invariant Retina
Source: Neuron. 2012 Sep 6;75(5):786–98. doi: 10.1016/j.neuron.2012.06.033 (PMC3485567; doi:10.1016/j.neuron.2012.06.033)
Supplement: Document S1. Figures S1–S4 and Supplemental Experimental Procedures [file mmc1.pdf]

Neuron, Volume *75*

Supplemental Information

How Variable Clones Build an Invariant Retina

Jie He, Gen Zhang, Alexandra D. Almeida, Michel Cayouette, Benjamin D. Simons, and William A. Harris

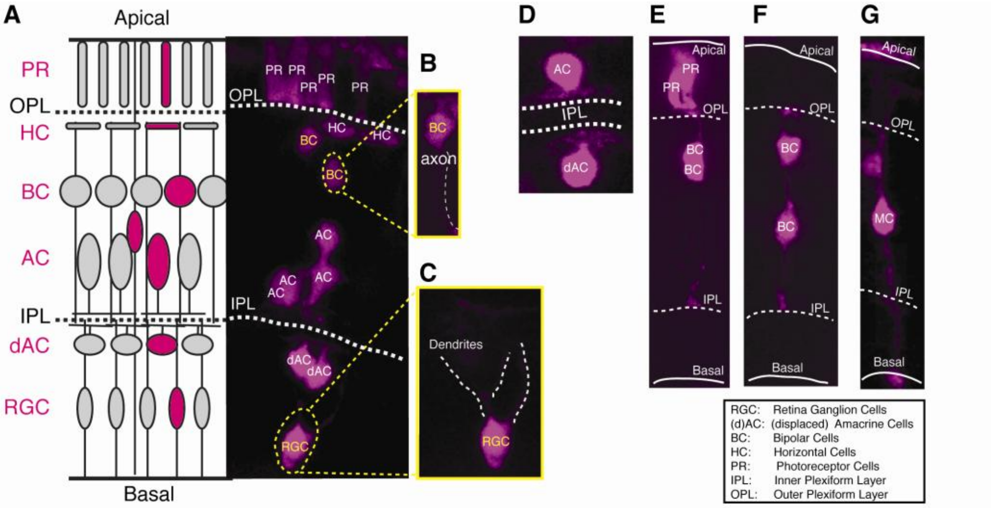

Figure S1. Retinal cell type identification. Distinct cell types are identified by the layer position and cell morphorlogy, which could be unambiguously examined in 3D reconstruction of a clone, as shown in the Movie S1. (A) A schematic digram of the anatomical retinal structure (left) and a representative retinal clone expressing the photoconverted Kaede (right). (B,C) Zoomed BC and RGC in the clone (A, right). (D) An example of an AC and a displaced AC. (E) An example of two PRs and two BCs. (F) An example of two BCs. (G) An example of one MC.

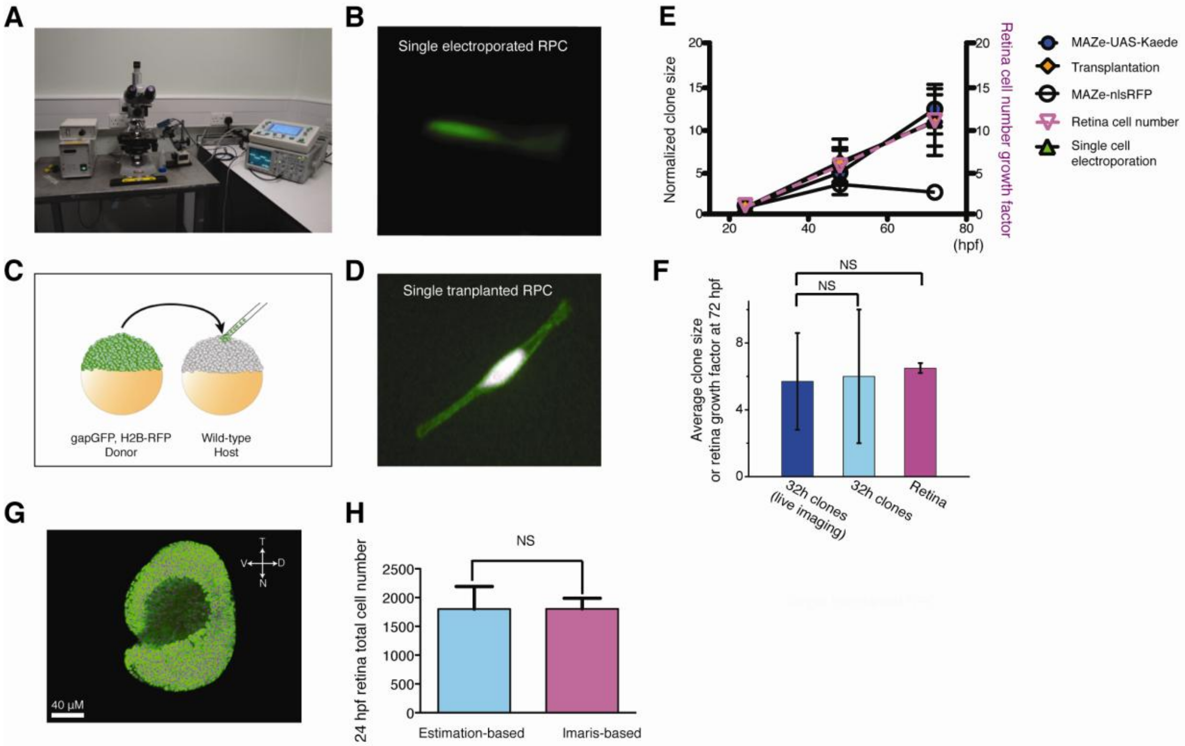

Figure S2. *In vivo* labeling of single retinal progenitor cells (RPCs). (A) A picture showing the setup for *in vivo* single-cell electroporation. (B) A single RPC electroporated with Dextran-Alexa 488 dye at 24 hpf. (C) A schematic diagram showing cell transplantation between a donor and host embryo. (D) Single RPC expressing gapGFP and H_2B-RFP in a 24 hpf host retina following transplantation. (E) Plot showing that the clones generated by these different labeling methods grow in size in an indistinguished manner, which is further comparable to total cell number growth of retina tissue over development. However, significant cell loss was observed in MAZe-nlsRFP expressing clones, in which the average clone size decreases after 48 hpf, finally dropping to 2.8 cells at 72 hpf (n=395). While, it is unclear why MAZe-nlsRFP labelled cells appear to die after 48hpf, this anomalous result emphasizes why it is critical to test the representativeness of clones induced by any labeling method if one is to conduct a rigorous clonal analysis of tissue development. (F) The size of 32 hpf-induced clones in live imaging is comparable to that of the 32h clones in the stain and fix experiment, and retina tissue growth.Values represent mean ± SD (n=71, 169, 4 for live imaging clones, 32h fixed clones and retina, NS, not significant). (G) Representative 3D reconstruction of a 24 hpf retina in which all the cell nucleus were recognized (Magenta color) by creating surface for every nucleus using Imaris. (H) Bar graph showing the total cell number of the 24 hpf retina measured by the cell numberestimation protocol (left, Estimation-based) described in Method and the discontinuous surface-based approach (right, Imaris-based).Values represent mean ± SD (n=8, cell estimation-based protocol; n=4, Imaris-based method; NS, no significance; $p > 0.05$; Students' t test). N: nasal; T: temporal; V: ventral; D: dorsal. All the cells express H_2B-GFP.

A

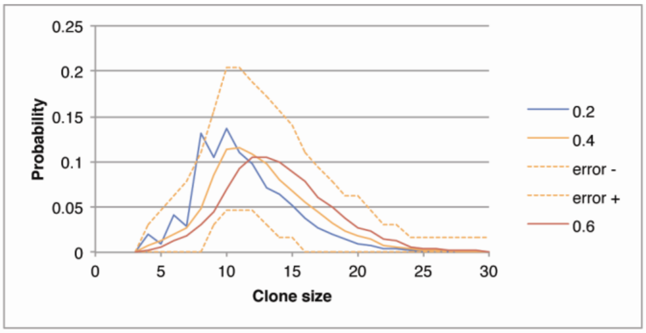

B

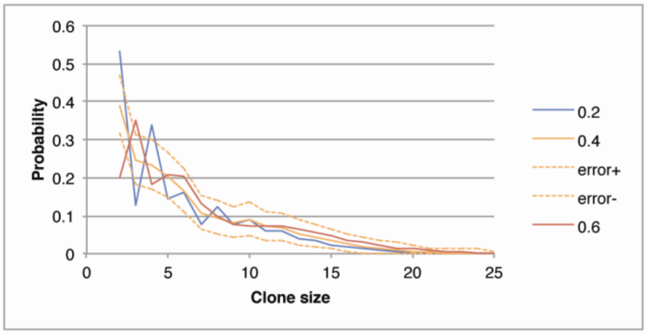

Figure S3 Variation of model predictions for induction at (A) 24hpf and (B) 32hpf and imaging at 72hpf. Here we vary PD division proportion during the intermediate phase. The orange line, PD=0.4, is found to fit the data best and is used throughout the text. We show the 95% plausible interval for PD=0.4 in dashed lines (error+ and error-), as proxy for the data. Note that the strongest constraint comes from the 32hpf data.

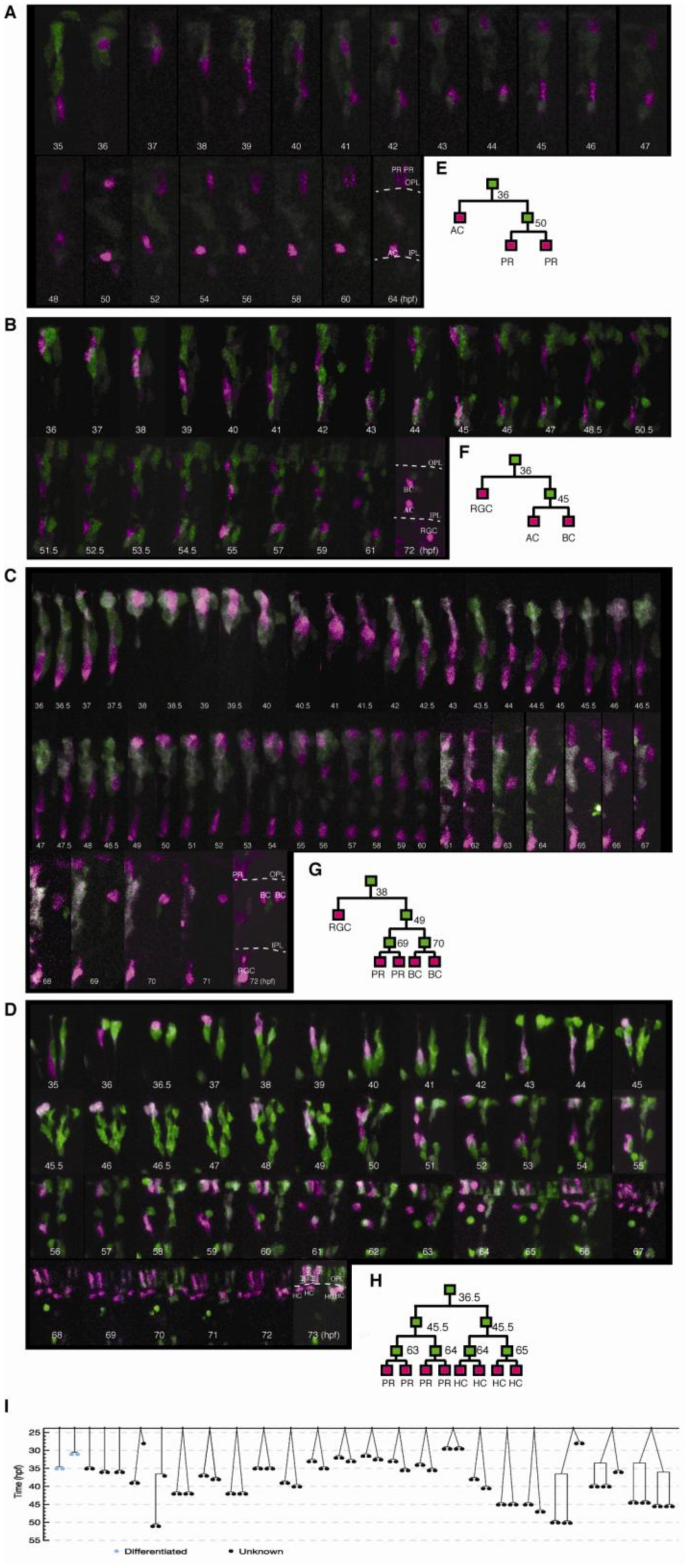

Figure S4. Development of retinal clones derived from single RPCs photoconverted at 32 hpf. (A-D) Time series of two 3-cell, one 5-cell, one 8-cell clones generated from single RPCs (in Magenta), in which every division and cell fate are recorded (shown in the schematic reconstructed trees in (E-H). (I) Time lapse of retinal clones from 24 to 48 hpf. Shows reconstructed lineages from early (24-48 hpf)live imaging movies using MAZe fish. Dashed lines show the clones which come from labelled RPCs that had already divided once by the time we began imaging.

Table S1.

(See accompanying Excel file.)

Here, we explain how we estimate the density of cells from confocal images. An obvious way to do so is to count the number of cells in a given volume and obtain a density as the ratio. However, this procedure only gives the correct answer if we are careful to count a cell as *inside* the prescribed volume if and only if its *centre* is inside. In practice, this can be difficult to ascertain, especially in the case when our volume is one single confocal z-slice (or generally where a slice is close to being infinitely thin); furthermore, in that case, the number of cell centres actually inside the volume is close to zero.

This documents describes the necessary corrections.

It is instructive to start with in 2D, using a symmetric shape such as a circle. Below, we have a typical area (analogue of volume in 3D) with some cells. Here we use the criterion that a cell is counted if it appears *at all* within the area. Compared to the *correct* criterion

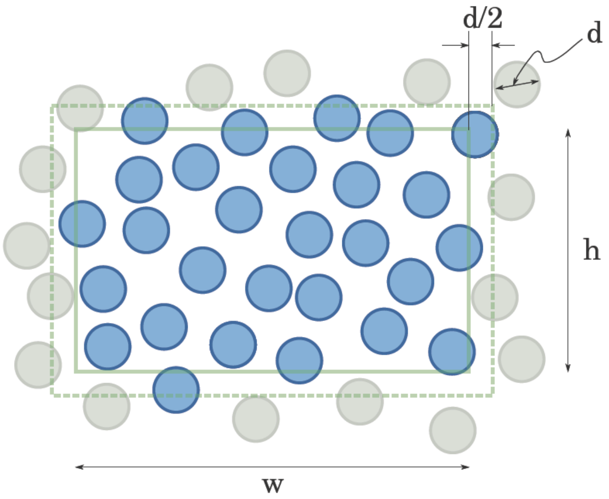

where we only count cells if their centres are inside, this causes an overcounting. We can correct for this by noticing that the effect is to count all cells with centres within an enlarged area with width $w + r$ and $h + r$. Thus we get a formula

$$\rho_{2\mathrm{D}} = \frac{N}{(w+d)(h+d)},$$

where N is the number of counted cells (28 in the above picture).
Now let us take the analogue of a confocal z-slice in 2D: a line through the cells. Again, we count the cells if any part of it appear in the line. Our formula above has no problems with this

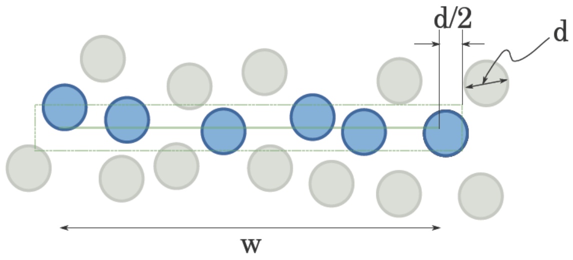

case, simply using $h = 0$:

$$\rho_{2\mathrm{D}} = \frac{N}{(w+d)d}.$$

We now focus on the specific case of a confocal slice in 3D. Generalising from above, if we have spherical cells of diameter d, and a confocal slice of thickness t and area $w \times h$, the density is straightforwardly:

$$\rho_{3\mathrm{D}} = \frac{N}{(w+d)(h+d)(t+d)}.$$

The only complication is if the cells are not isotropic. In general, this then becomes very complex, but in the special case that the plane is aligned with the axes, we can still perform the correction by measuring the diameter of the cell within ($d||$) and transverse ($d\perp$) to the plane. In that case,

$$\rho_{3\mathrm{D}} = \frac{N}{\left(w+d_{||}\right)\left(h+d_{||}\right)(t+d_{\perp})}.$$

Often, the slice taken is large in area to any single cell, but rectangular; as long as the area does not have a large aspect ratio, we may use an approximation which introduces very little error (much smaller than measurement errors in the various diameters, say):

$$\rho_{3\mathrm{D}} = \frac{N}{\left(A+p\times d_{||}\right)(t+d_{\perp})},$$

where A and p are the area and perimeter of the z-slice.
