# Supplementary figures and images for: How Variable Clones Build an Invariant Retina
Source: Neuron. 2012 Sep 6;75(5):786–98. doi: 10.1016/j.neuron.2012.06.033 (PMC3485567; doi:10.1016/j.neuron.2012.06.033)

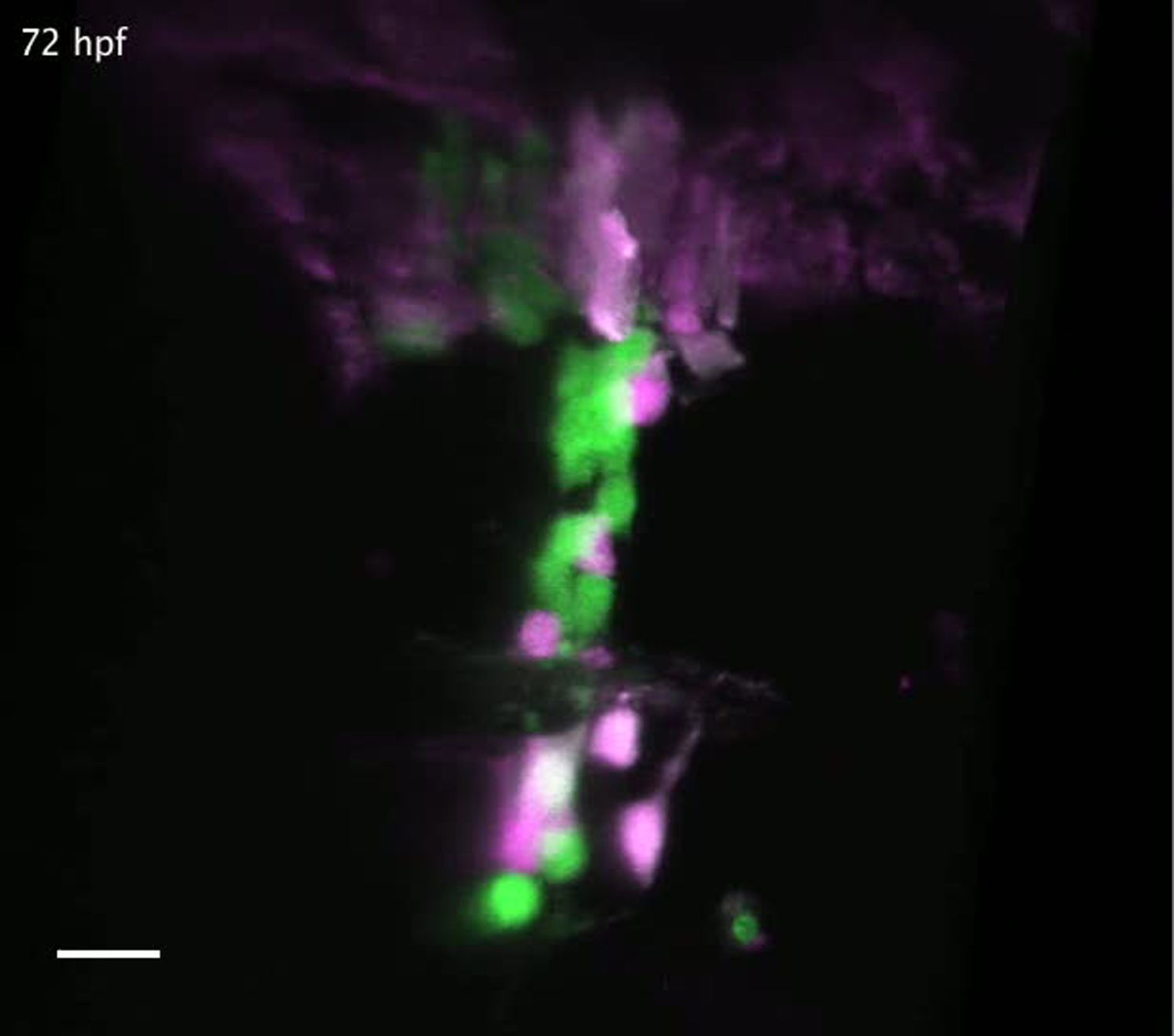

Supplement: Movie S1. Sister Clones in 3D — Three-dimensional animation of a pair of retina sister clones (in green and magenta) at 72 hpf, which are generated by photoconversion of a single retina progenitor cell from a Kaede-expressing two-cell clone at 24 hpf. Scale bar represents10 μm. [file mmc3.jpg]

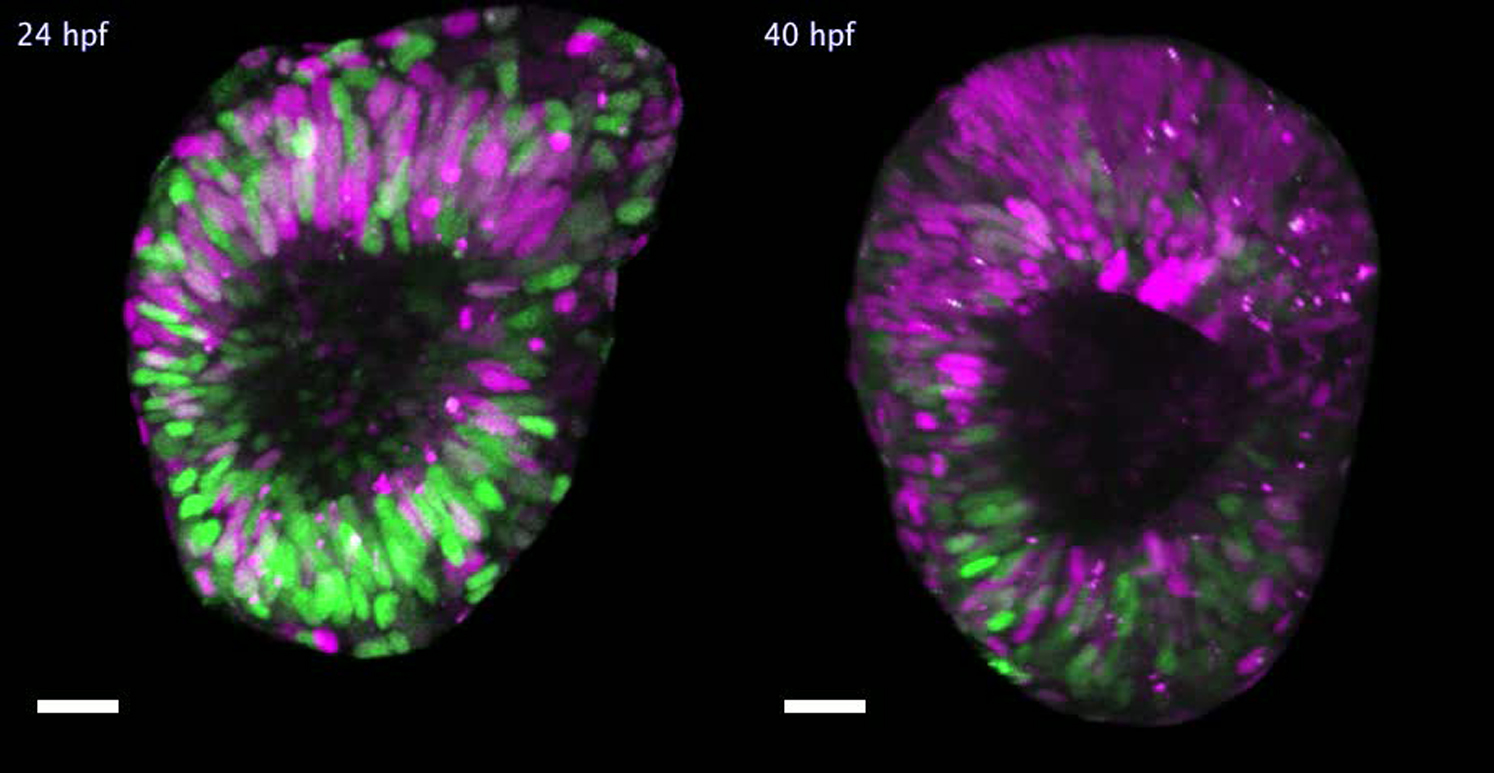

Supplement: Movie S2. mAG-zGEM in 3D — Three-dimensional animation of the retina with the expression of mAG-zGem (green) and H2B-RFP (magenta, mRNA-injected at the one-cell stage) at 24 and 40 hpf, showing retina nasal zone expands its size prior to the temporal zone, while the nasal progenitors are leaving the cell cycle (indicated by the loss of mAG-zGem) earlier than those from the temporal zone. Scale bar represents 25 μm. [file mmc4.jpg]

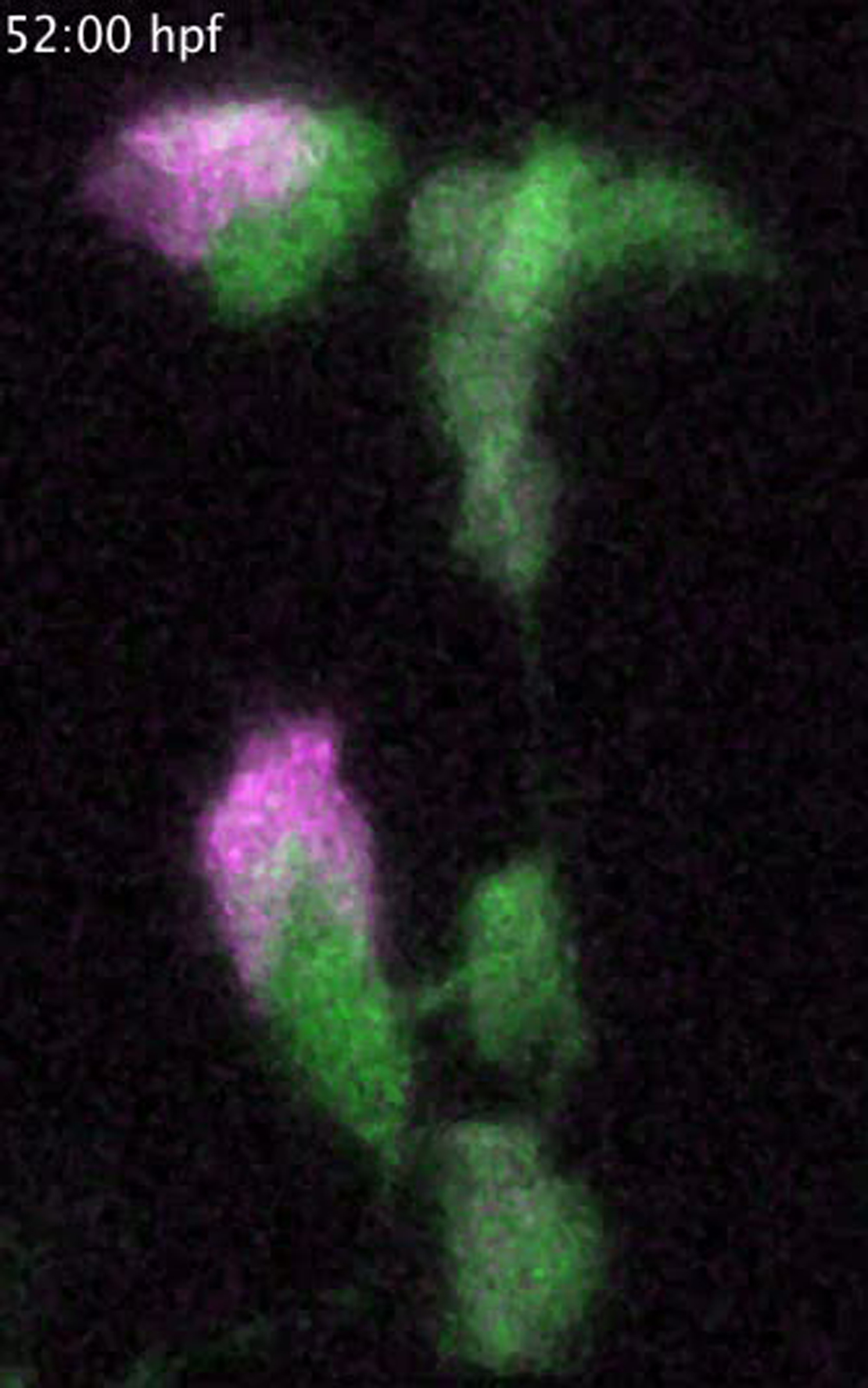

Supplement: Movie S3. Time Lapse of Eight-Cell Clone — Time lapse of an eight-cell clone (in Figures 4A and 4B) developed from a single RPC (magenta) from 35 hpf throughout 73 hpf. [file mmc5.jpg]
